# Stenosis coexists with compromised α1-adrenergic contractions in the ascending aorta of a mouse model of Williams-Beuren syndrome

**DOI:** 10.1038/s41598-020-57803-3

**Published:** 2020-01-21

**Authors:** Francesc Jiménez-Altayó, Paula Ortiz-Romero, Lídia Puertas-Umbert, Ana Paula Dantas, Belén Pérez, Elisabet Vila, Pilar D’Ocon, Victoria Campuzano

**Affiliations:** 1 Departament de Farmacologia, de Terapèutica i de Toxicologia, Facultat de Medicina, Institut de Neurociències, Universitat Autònoma de Barcelona, Bellaterra, Spain; 2 Departament de Ciències Experimentals i de la Salut, Universitat Pompeu Fabra, Barcelona, Spain; 3 Centro de Investigación Biomédica en Red de Enfermedades Raras (CIBERER), ISCIII, Barcelona, Spain; 4 Group of Atherosclerosis and Coronary Disease, Institut Clinic del Torax, Institut d’Investigaciones Biomédiques August Pi i Sunyer (IDIBAPS), Barcelona, Spain; 5 Departamento de Farmacología, Facultad de Farmacia, Universitat de València, Valencia, Spain; 6 Present Address: Departament de Biomedicina, Universitat de Barcelona; Centro de Investigación Biomédica en Red de Enfermedades Raras, ISCIII, 08036 Barcelona, Spain

**Keywords:** Valvular disease, Aortic diseases

## Abstract

Williams-Beuren syndrome (WBS) is a rare disorder caused by a heterozygous deletion of 26–28 contiguous genes that affects the brain and cardiovascular system. Here, we investigated whether WBS affects aortic structure and function in the complete deletion (CD) mouse model harbouring the most common deletion found in WBS patients. Thoracic aortas from 3–4 months-old male CD mice and wild-type littermates were mounted in wire myographs or were processed for histomorphometrical analysis. Nitric oxide synthase (NOS) isoforms and oxidative stress levels were assessed. Ascending aortas from young adult CD mice showed moderate (50%) luminal stenosis, whereas endothelial function and oxidative stress were comparable to wild-type. CD mice showed greater contractions to KCl. However, α1-adrenergic contractions to phenylephrine, but not with a thromboxane analogue, were compromised. Decreased phenylephrine responses were not affected by selective inducible NOS blockade with 1400 W, but were prevented by the non-selective NOS inhibitor L-NAME and the selective neuronal NOS inhibitor SMTC. Consistently, CD mice showed increased neuronal NOS expression in aortas. Overall, aortic stenosis in CD mice coexists with excessive nNOS-derived NO signaling that compromises ascending aorta α1-adrenergic contractions. We suggest that increased neuronal NOS signaling may act as a physiological ‘brake’ against the detrimental effects of stenosis.

## Introduction

Williams-Beuren syndrome (WBS) [OMIM 194050] is a rare congenital multisystem disorder caused by a recurrent heterozygous deletion of 26–28 contiguous genes on chromosome band 7q11.23^1^. This syndrome affects males and females equally with a prevalence that is estimated to range between 1/7,500 and 1/10,000^2^. Mainly due to elastin (*ELN*) haploinsufficiency, this condition is commonly characterized by cardiovascular alterations that can occur early in life^3^, can evolve into potentially serious complications, and are the main cause of death in WBS patients^1,4^. Supravalvular aortic stenosis is the most frequent cardiovascular anomaly, affecting approximately 70% of patients^1,5^, and it is a potentially life-threatening condition^6,7^. Surgery may be required and drug treatments for this disorder and other cardiovascular manifestations in WBS generally not differ from that in the general population^8^. Therefore, a thorough analysis of the pathophysiological mechanisms of aortic disease in WBS is needed to define more safe and effective personalized therapies.

Mouse models of elastin deficiency have provided valuable insights into the mechanisms responsible for the development of aortic anomalies in WBS. Thus, partial rescue of *Eln*^−/−^ by transgenic expression of human *ELN*^9,10^ is sufficient to cause a similar aortic phenotype to typical WBS patients^11–15^. However, there is increasing evidence that other genes besides *Eln* may be relevant in modulating the WBS cardiovascular phenotype. Various mouse models carrying chromosome microdeletions affecting the WBS critical region have been generated to mimic the molecular defects present in patients^16,17^. Mice carrying a heterozygous distal deletion (DD) (from *Limk1* to *Trim*50) are hypertensive, show increased aortic oxidative stress levels, cardiac hypertrophy, decreased aortic compliance, and histological alterations characterized by aortic wall thickening and fragmentation of elastin lamellar units^18,19^. However mice carrying a complete deletion (CD) (from *Gtf2i* to *Fkbp6*) show mild hypertension and cardiac hypertrophy at 8 months of age^17^. Notably, aortic function has never been explored in the ascending aorta of either CD mice or other mouse models carrying WBS chromosome microdeletions. The identification of early aortic dysfunction in WBS may help predict and prevent disease-related cardiovascular problems.

Previous evidence suggests a role of aortic valve endothelium in the maintenance of valvular homeostasis^20^. The endothelium releases a number of vasoactive substances including nitric oxide (NO), which is a signaling molecule that has many beneficial and sometimes detrimental effects on cardiovascular function^21,22^. NO plays an important role in the decreased ability of a vessel to respond to persistent stimulation by α1-adrenoceptor agonists, a process called ‘NO-mediated desensitization’^23^. Moreover, in aorta, a modulatory role of neuronal NOS (nNOS) associated to activation of the α1_A_-adrenoceptor subtype has been proposed as a physiological ‘brake’ against the detrimental effects of excessive adrenergic vasoconstriction^24^. Remarkably, an impairment of NO responsiveness is reported in patients with aortic stenosis^25–27^, and a proportion of WBS patients manifest higher levels of oxidative stress^28^; effects that might be associated with aortic dysfunction. Deep understanding of development of functional aortic anomalies in animal models of WBS and exploration of the mechanisms potentially involved would identify proper targets for human therapy.

In the present study, we provide the first comprehensive assessment of thoracic aorta structure and reactivity in young CD mice, a mouse model carrying the most common deletion found in WBS patients, which often manifest cardiovascular disease early in life. We conclude that ascending aortas from young CD mice show stenosis and impaired phenylephrine-induced α1-adrenergic contractions because of a NO-mediated desensitization.

## Methods

### Animals

Three- to four-months old male heterozygous CD (n = 25) mice and age-matched wild-type (WT, n = 26) littermates were used. Genotyping was made as previously reported^29^. Mice were housed according to institutional guidelines (constant room temperature at 22 °C, 12 h: 12 h light-dark cycle, 60% humidity, and access to food and water *ad libitum*). Experimental procedures were approved by the Ethical Committee of the Parc de Recerca Biomèdica de Barcelona (PRBB, Barcelona, Spain). The PRBB has Animal Welfare Assurance (#A5388–01, Institutional Animal Care and Use Committee approval date 05/08/2009), granted by the Office of Laboratory Animal Welfare (OLAW) of the US National Institutes of Health. The research conforms to the European Commission Directive 86/609 CEE Art. 21 (1995) and the Guide for the Care and Use of Laboratory Animals published by the US National Institutes of Health (NIH Publication No. 85–23, revised 1996). All experiments of this study were carried out in a blinded fashion.

### Blood pressure measurements in conscious mice

Measurement of systolic blood pressure was performed in conscious WT and CD mice using the tail-cuff method (NIPREM 645; Cibertec, Madrid, Spain). The average systolic blood pressure of each mouse was determined from six consecutive measurements after habituation, as described^30^.

### Tissue preparation

**Figure 1:**
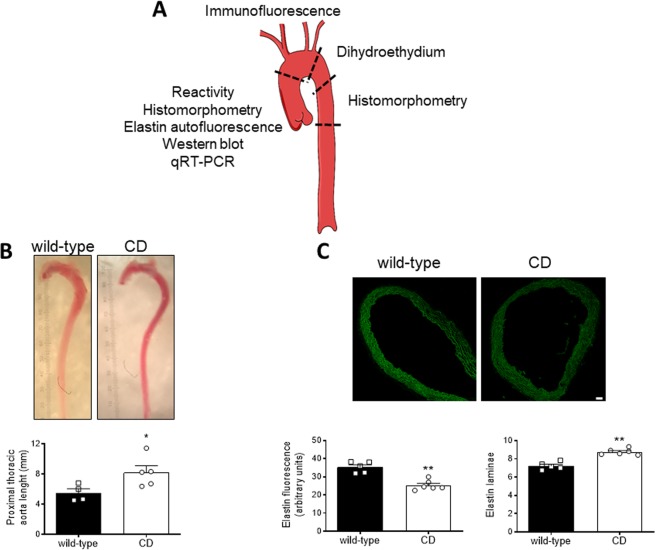
General characteristics of thoracic aortas from wild-type and CD mice. (**A**) Diagram illustrating the thoracic aorta segments used in the study. (**B**) Pictures showing representative *ex vivo* aortas and quantification of the proximal thoracic aorta length. Scale bar, 1 unit = 0.1 mm. Results are the mean ± SEM from wild-type (n = 4) and CD (n = 5) mice. *P < 0.05 by Student’s t-test. (**C**) Representative photomicrographs and quantification of elastin autofluorescence (green; bottom left) and number of elastin laminae (bottom right) in confocal microscopic ascending aorta sections. Scale bar, 40 μm. Results are the mean ± SEM from wild-type (n = 5) and CD (n = 6) mice. **P < 0.01 by Mann-Whitney *U* test.

Segments (Fig. 1A) of the ascending aorta (histomorphometry, reactivity, elastin autofluorescence, Western blot, and qRT-PCR), aortic arch (immunofluorescence and dihydroethidium), and the descending aorta (histomorphometry) were dissected free of fat and connective tissue and placed in ice-cold Krebs Henseleit solution gassed with a 95% O_2_–5% CO_2_ mixture. Aortic segments used for immunofluorescence studies were fixed with 4% phosphate buffered paraformaldehyde for 1 h, washed in three changes of phosphate buffered saline solution, and processed as described^31^. Plasma (200 μl) was collected after decapitation and was kept at −70 °C until the day of analysis of circulating superoxide anion levels by high-performance liquid chromatography (HPLC).

### *Ex vivo* gross examination of the proximal thoracic aorta

After dissection, images of the thoracic aorta were obtained *ex vivo* using a dissecting microscope (Leica, Wetzlar, Germany). The length of the proximal thoracic aorta (i.e. ascending aorta and aortic arch) was measured along its medial curvature from the ventricular-aortic junction to the distal aortic arch that finishes when the inner and outer curvature become parallel. Aortic length was measured from calibrated digital images using ImageJ 1.51j8 (National Institutes of Health, Bethesda, MD, USA) software.

### Measurement of elastin autofluorescence and number of elastin laminae

Total elastin content was studied in aortic cross-sections (14 μm-thick) based on the autofluorescent properties of elastin, as described^32^. Values of fluorescence intensity were estimated as a measure of elastin concentration, following the assumption that the concentration of elastin has a linear relationship with fluorescence intensity^33^. All of the images were taken using a laser-scanning confocal microscope (×20 objective; Leica TCS SP5, Manheim, Germany) under identical conditions of zoom (×1), laser intensity, brightness, and contrast. Quantitative analysis of elastin autofluorescence and number of elastin laminae was performed with ImageJ 1.51j8 software. The average intensity of fluorescence signal (expressed as arbitrary units) and the number of elastin laminae were measured in at least three rings from each animal.

### Aortic histomorphometry

Morphometric determination of aortic vessel and lumen area, and cross-sectional area (CSA) was performed using hematoxylin and eosin staining. Images were obtained with a Nikon Eclipse 80i microscope (×4 objective) and analyzed using ImageJ 1.51j8 software. The luminal and the vessel area, delimited by the internal elastic lamina and the external hematoxylin and eosin stained area, respectively, were calculated assuming a circle and applying the formula *l*^2^/4π, where *l* is the perimeter of the delimiting area, as described^34–36^. This correction circumvents inaccuracies in structural parameters calculations caused by the eventual collapse of the immersion-fixed arteries^34^. Wall thickness was calculated as follows: wall thickness = (*D*_e_ − *D*_i_)/2, where *D*_e_ and *D*_i_ are the external and internal diameter, respectively. *D*_*e*_ and *D*_*i*_ were extrapolated from the following formula: A = π·(D/2)^2^, where A is the vessel (*A*_e_) or the lumen (*A*_i_) area. The CSA was calculated as follows: CSA = *A*_e_ − *A*_i_. At least three sections from each artery/animal were measured.

### RNA preparation and gene expression quantification by qRT-PCR

RNA was extracted from ascending aortas using RNeasy Micro Kit (Qiagen, Hilden, Germany) according to the manufacturer’s instructions. cDNA was prepared from 1 µg of total RNA using random hexamers and SuperScript II RNase H reverse transcriptase (Invitrogen). The expression of *Adra1a* (α1_A_-adrenoceptors)*, Limk1* (a gene contained in the WBS commonly deleted region) and *Rps28* (internal control) was evaluated by quantitative PCR (qRT-PCR), as described^17^, using the appropriate primers (Supplementary Table S1). Each PCR was made with triplicates from two different RTs. The expression values were relativized according to the average expression of the WT animals for each gene.

### Analysis of circulating 2-hydroxyethidium (2-EOH)

Plasma levels of 2-EOH (Sigma-Aldrich, St. Louis, MO, USA) were assessed by HPLC with fluorescence detection, as a quantitative measure of plasma superoxide anion levels, as described^37–39^. 2-EOH present in the samples was quantified by comparing with a calibration curve based on the reaction xanthine-xanthine oxidase from the method described by Michalski and cols^40^.

### Measurement of aortic oxidative stress

The oxidative fluorescent dye dihydroethidium (DHE; Sigma-Aldrich) was used to evaluate *in situ* production of superoxide anion in non-fixed 14 μm-thick aortic sections, as described^41^. Quantitative analysis of DHE-derived fluorescence in images obtained using a laser-scanning confocal microscope (×20 objective; Olympus FluoView 1000; Olympus, Shinjuku, Tokio, Japan) was performed with ImageJ 1.51j8 software. At least three rings from each animal were measured and the results were expressed as arbitrary units.

### Aortic reactivity

Segments (2 mm) of the ascending thoracic aorta were set up on an isometric wire myograph (model 410 A; Danish Myo Technology, Aarhus, Denmark) filled with KHS (37 °C; 95% O_2_ and 5% CO_2_), as described^42^. Optimal tension was assessed in preliminary experiments by subjecting arterial segments to different resting tensions and challenging with 100 mM KCl^41,43^. The optimal tension of the ascending aorta was the same for WT and CD mice (5 mN). Therefore, the vessels were stretched to 5 mN, washed and allowed to equilibrate for 45 min. The tissues were contracted twice (10-min interval) with 100 mM KCl. After washing, vessels were left to equilibrate for a further 30 min before starting the experiments. Endothelial-dependent vasodilatations to acetylcholine (ACh; 10^−9^ to 10^−5^ M) were performed in phenylephrine-precontracted vessels to induce 70–100% of 100 mM KCl contraction, and thus produce a similar level of precontraction in either group. The use of thromboxane A_2_ mimetic 9,11-Dideoxy-9α,11α-methanoepoxyprostaglandin F_2α_ (U46619) to contract the vessels was discarded, because of U46619-induced precontractions greatly reduced ACh-induced vasodilatations. Agonist-induced contractile responses were studied by evaluating stimulation of α1-adrenoceptors with phenylephrine (10^−9^ to 3 × 10^−5^ M) or thromboxane A_2_ receptors with U46619 (10^−9^ to 3 × 10^−6^ M). The effects of the non-selective nitric oxide synthase (NOS) inhibitor Nω-nitro-l-arginine methyl ester (L-NAME; 3 × 10^−4^ M)^41^, the selective iNOS inhibitor 1400 W (10^−5^ M)^41^, and the selective nNOS inhibitor SMTC (10^−6^ M)^24^ were determined by adding each treatment 30 min before phenylephrine- or ACh-induced responses.

### Western blot

Similar amount of protein from each cell sample (20 µg) was resolved by SDS -PAGE on 4–12% gels and electroblotted onto nitrocellulose. Before immunoblotting, all membranes were labeled using a No-StainTM Protein Labeling Reagent (ThermoFisher scientific, Waltham, MA, USA) and fluorescent signal at 590 nm was used as a loading control. After washings and blocking with 2% casein solution in phosphate-buffered saline (PBS), membranes were incubated overnight at 4 °C in PBS with 0.1% (v/v) Tween 20 (PBST) containing 1% casein and specified primary antibodies as follows: 1:1000 mouse anti-eNOS (BD Biosciences, Franklin Lakes, NJ, USA, # 610297); 1:1000 rabbit anti-iNOS (ThermoFisher scientific, Waltham, MA, USA, #PA1-036); or 1:1000 mouse anti-nNOS (ThermoFisher scientific, Waltham, MA, USA, #37–2800). After incubation with horseradish peroxidase-labelled specific secondary antibodies in PBST containing 1% casein and additional washes, chemiluminescent signal was visualized by LAS4000 imaging system (Fujifilm, Barcelona, Spain). Densitometry of Western blots was performed using the ImageJ 1.51j8 Software. Data were normalized to corresponding values of total protein densitometry.

### Immunofluorescence

Fourteen-μm-thick aortic cross sections were obtained in a cryostat and were blocked with a 5% solution of bovine serum albumin (Sigma-Aldrich). Afterwards, sections were incubated with mouse monoclonal anti-eNOS (1:100; BD Biosciences, Franklin Lakes, NJ, USA, #610297), or a rabbit polyclonal anti-iNOS (1:50; ThermoFisher scientific, Waltham, MA, USA, #PA1-036) and anti-nNOS (1:100; ThermoFisher scientific, Waltham, MA, USA, #61-7000) antibodies at 37 °C, as described^41^. After washing, slides were treated with an anti-mouse (#715-165-150) or anti-rabbit (#711-165-152) Cy3^™^ secondary antibody (1:200; Jackson ImmunoResearch Laboratories Inc., West Grove, PA, USA). Sections were mounted with fluorescence mounting medium (Code S3023; Dako, Agilent, Santa Clara, CA, USA). Images were captured with an Olympus FluoView 1000 confocal system (×20 objective; Olympus, Shinjuku, Tokio, Japan). Several fluorescent regions between elastin laminae of the smooth muscle layer, and within the endothelium were delineated and averaged using ImageJ 1.51j8 software. Then, the global average intensity in at least three sections per animal was obtained.

### Statistical analysis

Data are presented as mean ± SEM of the number (n) of mice shown in figure legends. Vasodilator responses to ACh were expressed as a percentage of the previous tone generated by phenylephrine. Sigmoid curve fitting (variable slope) was performed by non-linear regression to obtain maximal responses (E_max_) and sensitivity (pEC_50_). Preliminary testing for normality was performed before using Mann-Whitney *U* test or Student’s t-test to compare the mean difference between two groups. Multiple t-test followed by Sidak-Bonferroni test as post hoc was used in the analysis of qRT-PCR data. Differences between concentration-response curves were also assessed by two-way repeated measures ANOVA followed by Bonferroni’s test as post hoc. The software GraphPad Prism 8 (GraphPad Software Inc., La Jolla, CA, USA) was used to run statistical analyses. A value of *P* < 0.05 was considered as statistically significant.

## Results

In agreement with a previous report^17^, young adult (3–4 months-old) CD mice showed significantly reduced (*P* < 0.001) body weight compared to WT mice (Supplementary Fig. S1A). There were no significant differences in systolic blood pressure between groups (Supplementary Fig. S1B).

### Young CD mice exhibited alterations in thoracic aorta morphology

**Figure 2:**
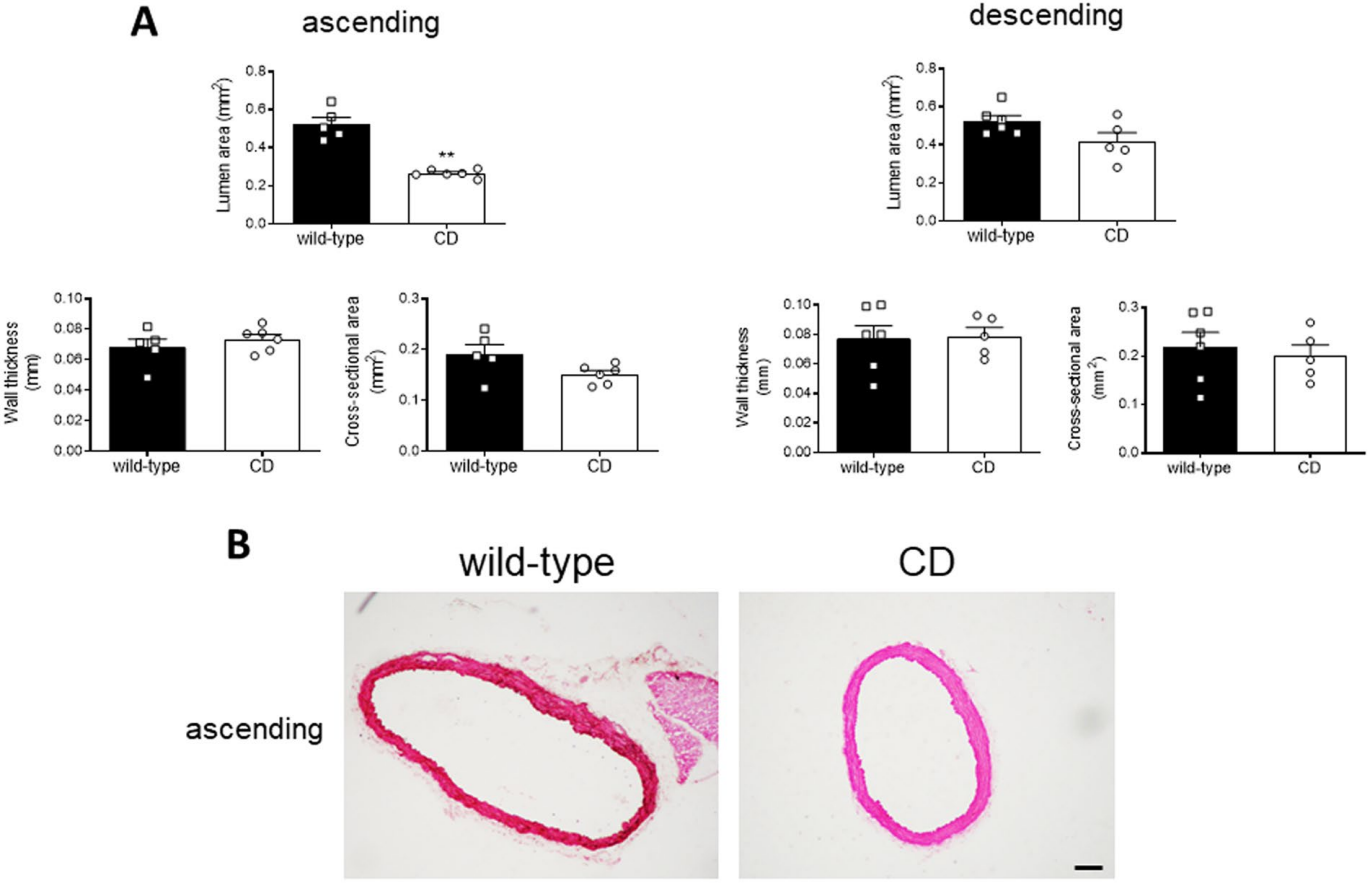
Structural characteristics of thoracic aortas from wild-type and CD mice. (**A**) morphometric analysis of the lumen area, wall thickness, and cross-sectional area of ascending and descending aorta histological sections stained with hematoxylin and eosin. Results are the mean ± SEM from wild-type (n = 5) and CD (n = 6) mice. **P < 0.01 by Mann-Whitney *U* test. (**B**) Representative photomicrographs of ascending aorta histological sections. Scale bar, 100 μm.

The proximal thoracic aorta, including the ascending aorta and the aortic arch, from CD mice was longer (*P* < 0.05; Fig. 1B) and showed reduced (*P* < 0.01) elastin autofluorescence and increased (*P* < 0.01) number of elastin laminae in the ascending aorta (Fig. 1C). Compared to WT mice, CD mice ascending aortas showed smaller vessel (*P* < 0.01; results not shown) and lumen (*P* < 0.01; Fig. 2A,B) areas, whereas wall thickness and CSA were not significantly altered (Fig. 2A,B). In contrast, descending aorta structure was similar between genotypes (Fig. 2A). These results suggest the presence of ascending aorta stenosis in CD mice.

### CD mice showed unaltered oxidative stress levels

**Figure 3:**
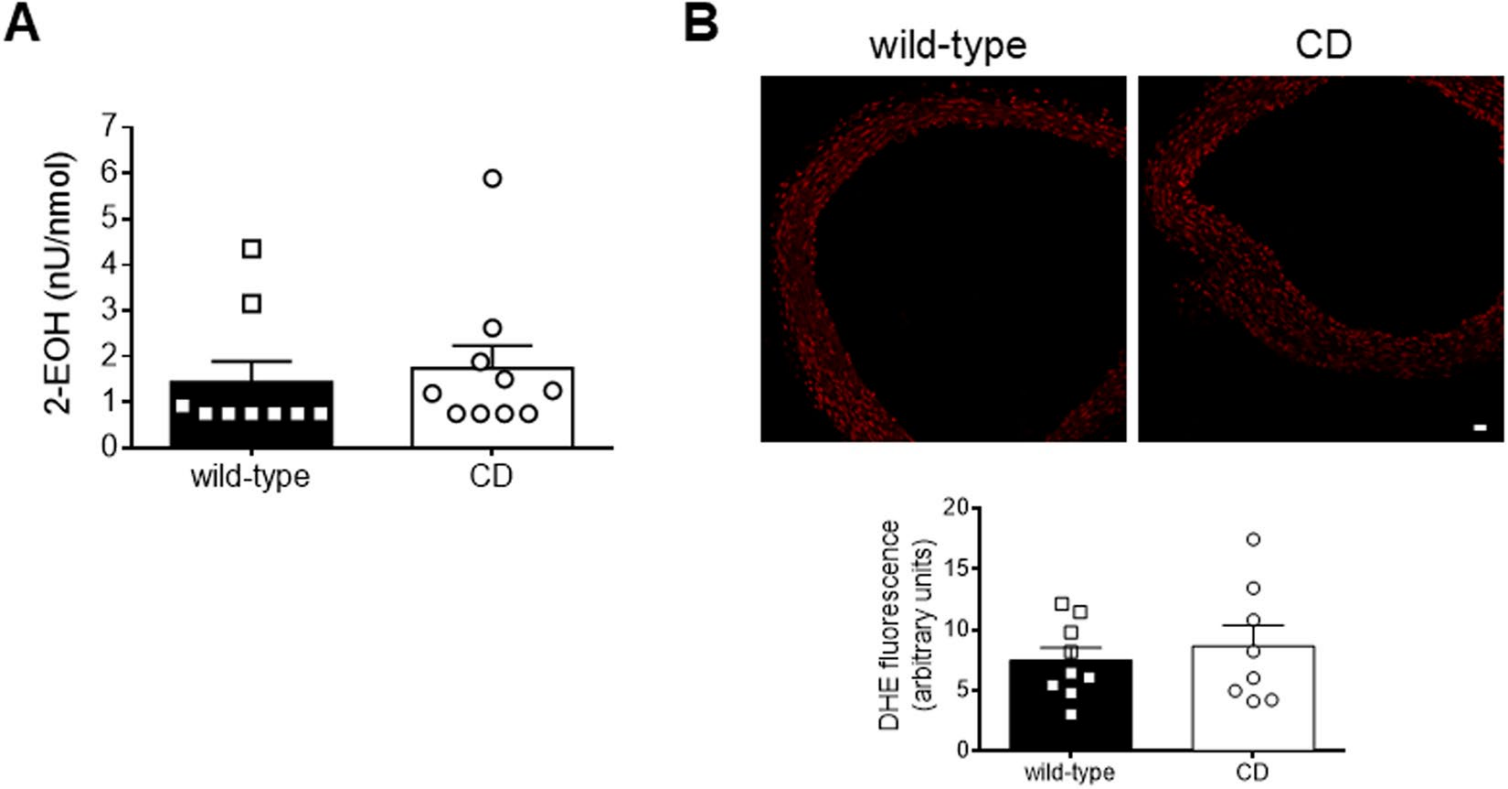
Oxidative stress levels in plasma and thoracic aortas from wild-type and CD mice. (**A**) Plasma levels of 2-hydroxyethidium (2-EOH). Results are mean ± SEM from wild-type (n = 9) and CD (n = 10) mice. (**B**) Representative photomicrographs and quantification of fluorescence (red) intensity of confocal microscopic sections labelled with the oxidative dye dihydroethidium (DHE). Scale bar: 20 µm. Results are the mean ± SEM from wild-type (n = 9) and CD (n = 8) mice.

Plasma levels of 2-EOH, an indirect measure of circulating superoxide anions^37,39^, were similar between genotypes (Fig. 3A). To assess *in situ* oxidative stress production, we evaluated aortic wall oxidative stress formation by studying DHE-derived fluorescence (Fig. 3B). Aortas from CD mice showed similar levels of DHE fluorescence compared to WT mice. Altogether, these data suggest that CD mice do not show altered levels of either circulating or aortic oxidative stress.

### Endothelial function was preserved in aortas from CD mice

**Figure 4:**
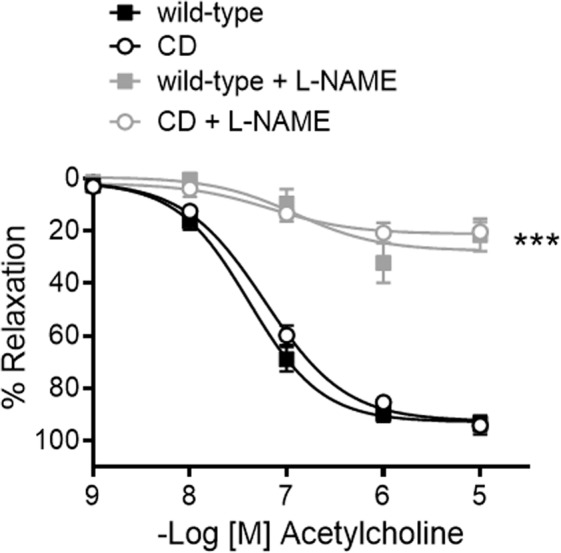
Endothelium-dependent relaxation and influence of nitric oxide synthase (NOS) inhibition in ascending aortas from wild-type and CD mice. Concentration-response curves to acetylcholine were evaluated in the absence and presence of the nonselective nitric oxide synthase inhibitor L-NAME (3 × 10^−4^ M). Results are the mean ± SEM from wild-type (n = 5–15) and CD (n = 4–13) mice. ***P < 0.001 by two-way ANOVA.

There were no differences in endothelium-dependent ACh-induced vasodilatation in the aorta of CD and WT mice (Fig. 4; Table 1). The non-selective NOS inhibitor L-NAME (3 × 10^−4^ M) greatly decreased (*P* < 0.001) ACh-induced vasodilatation in either group (Fig. 4) indicating that in both genotypes the ACh-induced vasodilatation is mediated by NO signaling. These results suggest that endothelial function is preserved in CD mice.

**Table 1:** *p*EC_50_ and E_*max*_ values for phenylephrine (PHE)- and U46619-induced contraction (mN) and acetylcholine (ACh)-induced relaxation (%) in the ascending aorta from wild-type and CD mice.

	wild-type	CD
***p*EC**_***50***_		
PHE	6.57 ± 0.09 (15)	6.42 ± 0.20 (13)
U46619	6.92 ± 0.07 (3)	7.15 ± 0.09 (3)
ACh	7.38 ± 0.08 (15)	7.23 ± 0.07 (13)
**E**_***max***_		
PHE	4.19 ± 0.20 (15)	2.64 ± 0.28 (13)***
U46619	16.50 ± 0.81 (3)	14.80 ± 0.83 (3)
ACh	92.74 ± 2.53 (15)	92.49 ± 2.48 (13)

Data are shown as mean ± SE. The number of animals is shown in parentheses. ***P < 0.001 versus wild-type by unpaired Student’s t-test.

### Contractile responses were altered in aortas from CD mice

**Figure 5:**
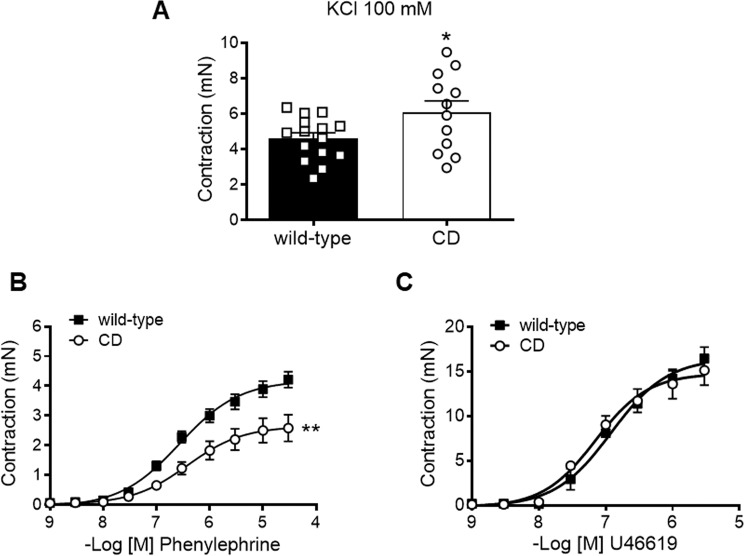
Contractile responses in ascending aortas from wild-type and CD mice. (**A**) KCl 100 mM-induced contraction. (**B**) Concentration-response curves to phenylephrine. (**C**) Concentration-response curves to U46619. Results are the mean ± SEM from wild-type (n = 3–15) and CD (n = 3–13) mice. *P < 0.05 by Student’s t-test; **P < 0.01 by two-way ANOVA.

Contractile responses to KCl (100 mM) were significantly higher (*P* < 0.05) in CD compared to WT aortas (Fig. 5A). Despite enhanced KCl responses, concentration-dependent contractions to phenylephrine were markedly decreased in the aorta of CD compared to WT mice (Fig. 5B), since the E_max_ of CD mice aortas was significantly lower (*P* < 0.001) than that of WT aortas (Table 1). In contrast, contractions to U46619 were similar between groups (Fig. 5C; Table 1). These findings suggest that there is a lower aortic vasoconstriction specific to α1-adrenergic-mediated responses.

### NO-mediated modulation of phenylephrine contractions was increased in CD mice

**Figure 6:**
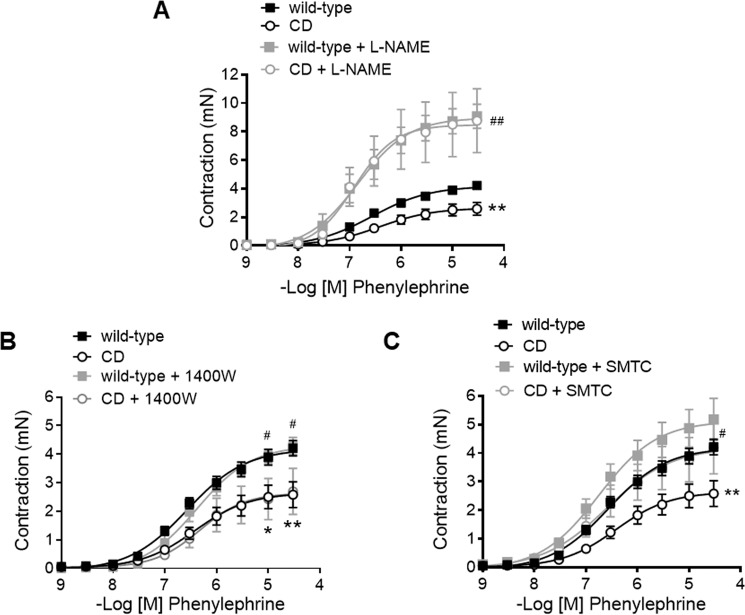
Influence of nitric oxide synthase (NOS) inhibition on ascending aortas phenylephrine contractions from wild-type and CD mice. Concentration-response curves to phenylephrine in the absence and presence of (**A**) the nonselective NOS inhibitor L-NAME (3 × 10^−4^ M), (**B**) the selective inducible NOS inhibitor 1400 W (10^−5^ M) and (**C**) the selective neuronal NOS inhibitor SMTC (10^−6^ M). Results are the mean ± SEM from wild-type (n = 4–15) and CD (n = 4–13) mice. *P < 0.05, **P < 0.01 CD vs wild-type; ^#^*P* < 0.05, ^##^*P* < 0.01 treated versus non-treated by two-way ANOVA.

In the thoracic aorta, phenylephrine contractile responses are counteracted by NO release, as evidenced by treatment of arterial rings with L-NAME (3 × 10^−4^ M), which enhanced (i.e. higher E_max_; Fig. 6A, Table 2) the concentration-response curve to phenylephrine in both WT and CD mice. Notably, L-NAME treatment reversed the impairment of phenylephrine contractions observed in CD mice, as in these conditions, no contractile differences were observed between groups (Fig. 6A, Table 2). To elucidate the source of NO involved in contractile differences, we then evaluated the effects of the selective iNOS inhibitor 1400 W (10^−5^ M) that had no effect on phenylephrine-induced contractions in either group (Fig. 6B, Table 2). In contrast, treatment with SMTC (10^−6^ M), a selective nNOS inhibitor, increased (*P* < 0.05) the E_max_ of phenylephrine contractions in WT and CD mice, an effect that prevented compromised aortic contractions in CD mice (Fig. 6C, Table 2). Taken together, these results suggest that NO-mediated negative feedback underlies impaired phenylephrine contractions in CD mice, an effect that depends on nNOS activation.

**Table 2:** *p*EC_*50*_ and E_*max*_ values for phenylephrine-induced contraction (mN) in the ascending aorta from wild-type and CD mice.

	wild-type				CD			
	Control	+L-NAME 3 × 10^–4^ M	+1400 W 10^–5^ M	+SMTC 10^–6^ M	Control	+L-NAME 3 × 10^–4^ M	+1400 W 10^–5^ M	+SMTC 10^–6^ M
***p*EC**_***50***_	6.57 ± 0.09 (15)	6.84 ± 0.15 (5)	6.38 ± 0.10 (4)	6.75 ± 0.18 (6)	6.42 ± 0.20 (13)	6.91 ± 0.25 (4)	6.33 ± 0.28 (4)	6.60 ± 0.30 (5)
**E**_***max***_	4.19 ± 0.20 (15)	8.97 ± 0.59 (5)***	4.31 ± 0.24 (4)	5.12 ± 0.42 (6)*	2.64 ± 0.28 (13)^###^	8.47 ± 0.94 (4)***	2.64 ± 0.40 (4)^#^	4.19 ± 0.63 (5)*

Data are shown as mean ± SE. The number of animals is shown in parentheses. *P < 0.05, ***P < 0.001 versus Control; ^#^*P* < 0.05, ^###^*P* < 0.001 versus the same group in wild-type by unpaired Student’s t-test.

### Increased levels of α1_A_-adrenoceptor and nNOS were found in aortas from CD mice

**Figure 7:**
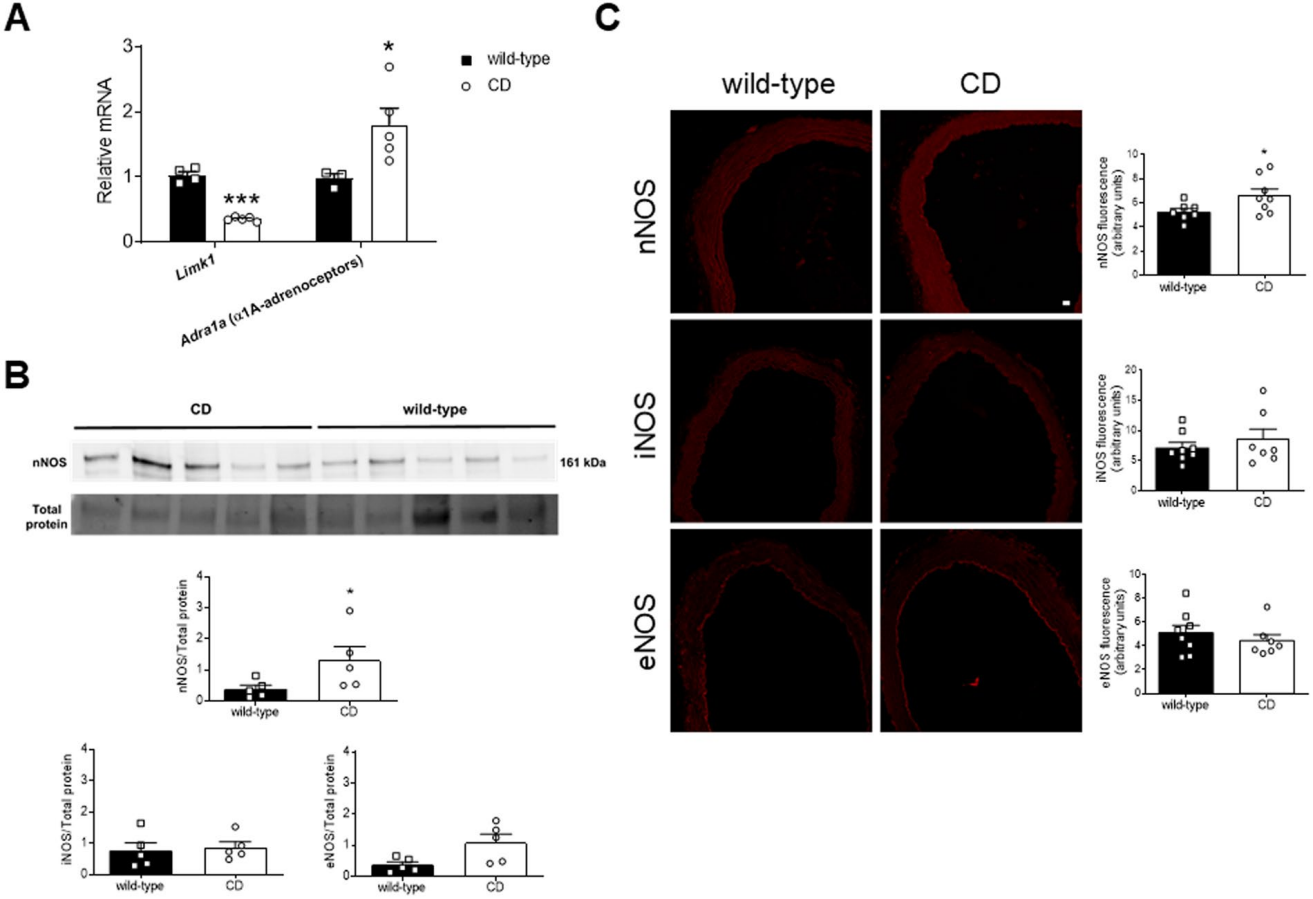
Genic and protein expression in thoracic aortas from wild-type and CD mice. (**A**) qPCR mRNA expression of *Limk1*, a gene contained in the Williams-Beuren syndrome critical region, and *Adra1a* (α1_A_-adrenoceptors). Results are the mean ± SEM from wild-type (n = 4) and CD (n = 5) mice. *P < 0.05; ***P < 0.01 by Multiple t-test with Sidak-Bonferroni. (**B**) Western blot analysis for nNOS protein expression. Bar graphs show the results of densitometric analyses from pooled data for nNOS, iNOS, and eNOS protein expression. The molecular weight (kDa) of the protein is shown on the right side of the blot. Total protein was used as a loading control. Results are the mean ± SEM from wild-type (n = 5) and CD (n = 5) mice. *P < 0.05 by Mann-Whitney *U* test. (**C**) Representative photomicrographs and quantification of immunofluorescence (red) for nNOS, iNOS, and eNOS in confocal microscopic aortic sections. Scale bar, 20 μm. Results are the mean ± SEM from wild-type (n = 8) and CD (n = 8) mice. *P < 0.05 by Student’s t-test.

A modulatory role of nNOS associated to activation of the α1_A_-adrenoceptor subtype in aorta has been proposed as a physiological ‘brake’ against the detrimental effects of excessive adrenergic vasoconstriction^24^. We observed an increase (*P* < 0.05) in aortic *Adra1a* (α1_A_-adrenoceptors) mRNA levels in CD compared to WT mice (Fig. 7A). On the other hand, aortic expression of nNOS was also increased (*P* < 0.05) in CD mice (Fig. 7B), and was detected in both endothelial and smooth muscle cell layers (Fig. 7C). The fluorescence signaling was more marked in CD mice, particularly in the endothelium (Fig. 7C). Consistently, quantitative analysis of fluorescence showed greater levels (*P* < 0.05) of nNOS expression in aortas from CD compared to WT mice (Fig. 7C). However, similar levels of iNOS (equally distributed in all layers of the aortic wall) and eNOS (mainly located in the endothelium layer) were observed in WT and CD mice (Fig. 7C; Supplementary Fig. S2).

## Discussion

Several mice models carrying chromosome microdeletions affecting the WBS critical region have been generated^16,17^. These models are crucial to study potential alterations in vascular responses associated with WBS, a significant question that has not yet been fully addressed. The present study examined ascending aorta structure and reactivity in WT compared to CD mice harbouring the most common deletion found in WBS patients^17,44^. The results show the presence of luminal stenosis and compromised contractile responses to α1-adrenoceptor activation associated with increased NO signaling in CD mice ascending aortas.

Approximately half of WBS patients develop hypertension^1,45^. This fact has been correlated with higher levels of oxidative stress due to increased expression of *Ncf1*^19,28^, which encodes the p47^phox^ subunit of the NADPH oxidase, the major source of superoxide anions in the vasculature^46^. However, a previous study of our group reported mild hypertension in 8-months old CD mice without increased levels of *Ncf1* in the heart and the aorta^17^, suggesting that CD mice may develop slight increases in blood pressure later in life. Notably, CD mice showed cardiac hypertrophy^17,44^ that was associated with increased levels of oxidative stress in the heart^44^. In the present study, young (3–4 months-old) CD mice did not show high systolic blood pressure nor greater levels of circulating or *in situ* aortic superoxide anions.

The proximal thoracic aorta (i.e. ascending aorta and aortic arch) from CD mice was longer and showed decreased elastin content, which is compatible with the elastin gene haploinsufficiency. In WBS, stenosis can occur diffusely, the most common sites being the thoracic aorta and the pulmonary vascular bed^5^. Supravalvular aortic stenosis is the most frequent cardiovascular anomaly, affecting approximately 70% of patients^1,5^. Aortic stenosis is a serious disease that if left untreated can increase the risk of suffering life-threatening complications, such as stroke^6^ and sudden death^7^. However, most experimental models of elastin haploinsufficiency do not show marked aortic luminal obstruction^47^. Notably, hemizygous deletion of the WBS critical region resulted in a moderate (50%) reduction in the ascending aorta lumen area compared to WT mice. Nevertheless, we did not measure histomorphometry in older animals to determine whether luminal obstruction subsequently progresses. Overall, young CD mice reproduce a crucial aspect of the human disorder, which is the presence of aortic stenosis close above the aortic root.

Relevant changes were observed in the functional characteristics of ascending aortas from CD mice. Thus, although endothelial-dependent relaxation to ACh was similar in either group, aortas from CD mice showed an increased contractile response to KCl-induced membrane depolarization together with compromised α1-adrenergic contractions to phenylephrine, despite contractions evoked by the thromboxane A_2_-analogue U46619 were unchanged. Notably, these attenuated α1-adrenergic contractions of WBS aortas were present in spite of higher contractile responses to KCl, which suggests that although the smooth muscle contractile phenotype is able to produce powerful contractions, there is a lower aortic vasoconstriction specific to α1-adrenergic-mediated responses. Given the importance of the α1-adrenergic control of vascular tone, the lower response to phenylephrine could represent an adaptive mechanism to counteract luminal stenosis. Although there is little information about the functioning of the autonomic nervous system in WBS patients, a previous study reported that WBS children show increases in both heart rate and wave reflection values during the night, which may suggest abnormal sympathetic nervous system hyperactivity in WBS^48^. The results of the present study suggest that this hyperactivity might be counteracted by a lower response to aortic α1-adrenoceptor activation. Nevertheless, additional evidence is needed to demonstrate that the results obtained in the present *ex vivo* study can be translated *in vivo*.

**Figure 8:**
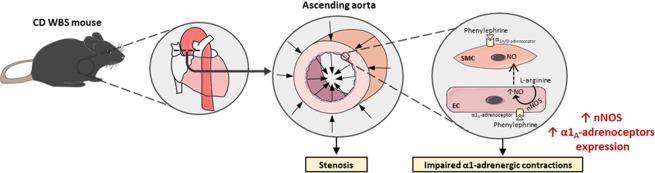
Diagram illustrating the main findings of the study and the proposed mechanism involved in the observed alterations. The ascending aorta of complete deletion (CD) mice shows stenosis and attenuated phenylephrine-induced contractions compared to wild-type mice. The present study suggests that phenylephrine, besides activating α1_A/D_-adrenoceptors located in smooth muscle cells (SMC) to induce contraction, could also stimulate endothelial cell (EC) α1_A_-adrenoceptors that could trigger the release of nitric oxide (NO) by endothelial neuronal nitric oxide synthase (nNOS)^24^, whose expression is increased in CD mice. This increased release of NO could be responsible for the impairment of α1-adrenoceptor-mediated contractions in the ascending aorta of CD mice. WBS, Williams-Beuren syndrome.

Endothelial NO can exert an opposing modulatory influence on α1-adrenoceptor agonist vasoconstriction^24,49,50^. eNOS plays an important role in the decreased ability of a vessel to respond to subsequent stimulation by the same agonist (NO-mediated desensitization)^23^. In addition, intense or sustained α1-adrenoceptor activity upregulates an endothelium-dependent nNOS pathway that attenuates α1-adrenoceptor-mediated contractions in the rat aorta^24^. This modulatory role of nNOS is associated with activation of the α1_A_-adrenoceptor subtype^24^ located in endothelial cells^51^. This effect can be physiologically relevant, as it might prevent significant increases in cardiac afterload. Therefore, our next objective was to determine if this mechanism was involved in the lower α1-adrenergic response observed in CD versus WT mice. Firstly, CD mice aortas showed increased aortic α1_A_-adrenoceptor expression, the subtype coupled to nNOS, and this change exhibited tissue specificity since it was not observed in the left ventricle (unpublished results). Secondly, our results pointed in the direction of a higher negative influence of NO on α1-adrenoceptor vasoconstriction in WBS, since compromised aortic contractions in CD mice were prevented by the non-selective NOS inhibitor L-NAME. In the vasculature, eNOS and nNOS isoforms of NOS are generally involved in regulatory or signaling pathways and play important roles in the modulation of vascular tone, whereas the iNOS isoform is related with inflammatory responses^21^. Inhibition of nNOS with SMTC, but not iNOS inhibition with 1400 W, significantly increased phenylephrine contractions in both strains, an effect that removed contractile impairments in CD mice. In addition, we detected an increased expression of nNOS in aortas from CD mice, whereas eNOS and iNOS expressions were similar to WT mice. Altogether, these results suggest a higher negative influence of NO on α1-adrenoceptor constriction in WBS mice, and propose that nNOS is a relevant NO source in these conditions (Fig. 8).

Augmented negative influence of NO in aortic adrenergic contractions might prevent excessive increases in cardiac afterload in the face of supravalvular aortic stenosis. Notably, impairment of tissue NO responsiveness is a main predictor of aortic stenosis development^25–27^, which provides additional evidence that NO may exert protective effects in WBS. Furthermore, clinical studies in patients with aortic stenosis suggest that nitrates can be used to lower blood pressure without excessive risk of reducing cardiac output or end-organ perfusion^52^, confirming a beneficial role for NO in this clinical setting.

In conclusion, our study reveals that ascending aorta obstruction coexist with functional alterations in a mouse model carrying the most common chromosomal deletion present in WBS patients. These findings support the clinical relevance of young CD mice to model moderate aortic stenosis associated with human WBS. The results show excessive nNOS-derived NO signaling that compromises ascending aorta α1-adrenergic contractions. Therefore, increased nNOS signaling may act as a physiological ‘brake’ against the detrimental effects of stenosis. We suggest that early detection and monitoring of thoracic aorta dysfunction would offer the potential for timely intervention in this syndrome.

## Supplementary information

Supplementary Information.

## Data Availability

The datasets generated during and/or analysed during the current study are available from the corresponding author on reasonable request.
