# Supplementary material for: Stenosis coexists with compromised α1-adrenergic contractions in the ascending aorta of a mouse model of Williams-Beuren syndrome
Source: Sci Rep. 2020 Jan 21;10:889. doi: 10.1038/s41598-020-57803-3 (PMC6972706; doi:10.1038/s41598-020-57803-3)
Supplement: Supplementary file 1 — Supplementary Information. [file 41598_2020_57803_MOESM1_ESM.pdf]

Stenosis coexists with compromised α1-adrenergic contractions in ascending aortas from a mouse model of Williams-Beuren syndrome

Francesc Jiménez-Altayó[1], Paula Ortiz-Romero[2,3+], Lídia Puertas-Umbert[1+], Ana Paula Dantas[4], Belén Pérez[1], Elisabet Vila[1], Pilar D'Ocon[5], Victoria Campuzano[2,3#]

[1]Departament de Farmacologia, de Terapèutica i de Toxicologia, Facultat de Medicina, Institut de Neurociències, Universitat Autònoma de Barcelona, Bellaterra, Spain; [2]Departament de Ciències Experimentals i de la Salut, Universitat Pompeu Fabra, Barcelona, Spain; [3]Centro de Investigación Biomédica en Red de Enfermedades Raras (CIBERER), ISCIII, Barcelona, Spain; [4]Group of Atherosclerosis and Coronary Disease, Institut Clinic del Torax, Institut d'Investigaciones Biomédiques August Pi i Sunyer (IDIBAPS), Barcelona, Spain; [5]Departamento de Farmacología, Facultad de Farmacia, Universitat de València, Valencia, Spain.

[#]Current address: Departament de Biomedicina, Universitat de Barcelona; Centro de Investigación Biomédica en Red de Enfermedades Raras, ISCIII, 08036 Barcelona, Spain.

Supplementary Table 1

Supplementary Table 1. Primer sequences used in qRT-PCR studies.

Gene	Sequence (5' →3')		Size	Location	Temperature (ºC)
***Adra1a* (α1$_A$-adrenoceptors)**	L	GAGAGAAGAAAGCCGCCAAG	237	exon2	61.5
	R	ACTGGATTCGCAGCACATTC		exon3	61.2
Limk1	L	CCTACCTCCATTCGATGAACA	262	exon 12	63.8
	R	CCAAAGGAAAACACGTCCA		exon 14	64.3
Rps28	L	TAGGGTAACCAAAGTGCTGGG	103	exon 1-2	67.9
	R	GACATTTCGGATGATAGAGCGG		exon 3	66.6

Supplementary Figure 1

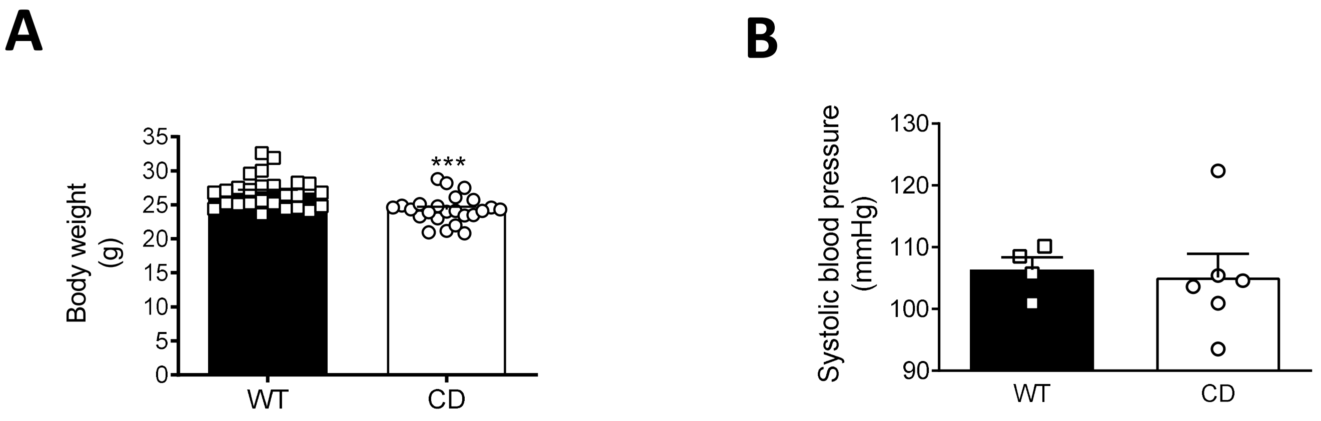

Supplementary Figure 1. Characteristics of wild-type (WT) and complete deletion (CD) mice. (A) Body weight and (B) systolic blood pressure in wild-type and CD mice. Results are the mean ± SEM from WT (n = 4-26) and CD (n = 6-24) mice. ****P* < 0.001 by Student's t-test (B).

Supplementary Figure 2

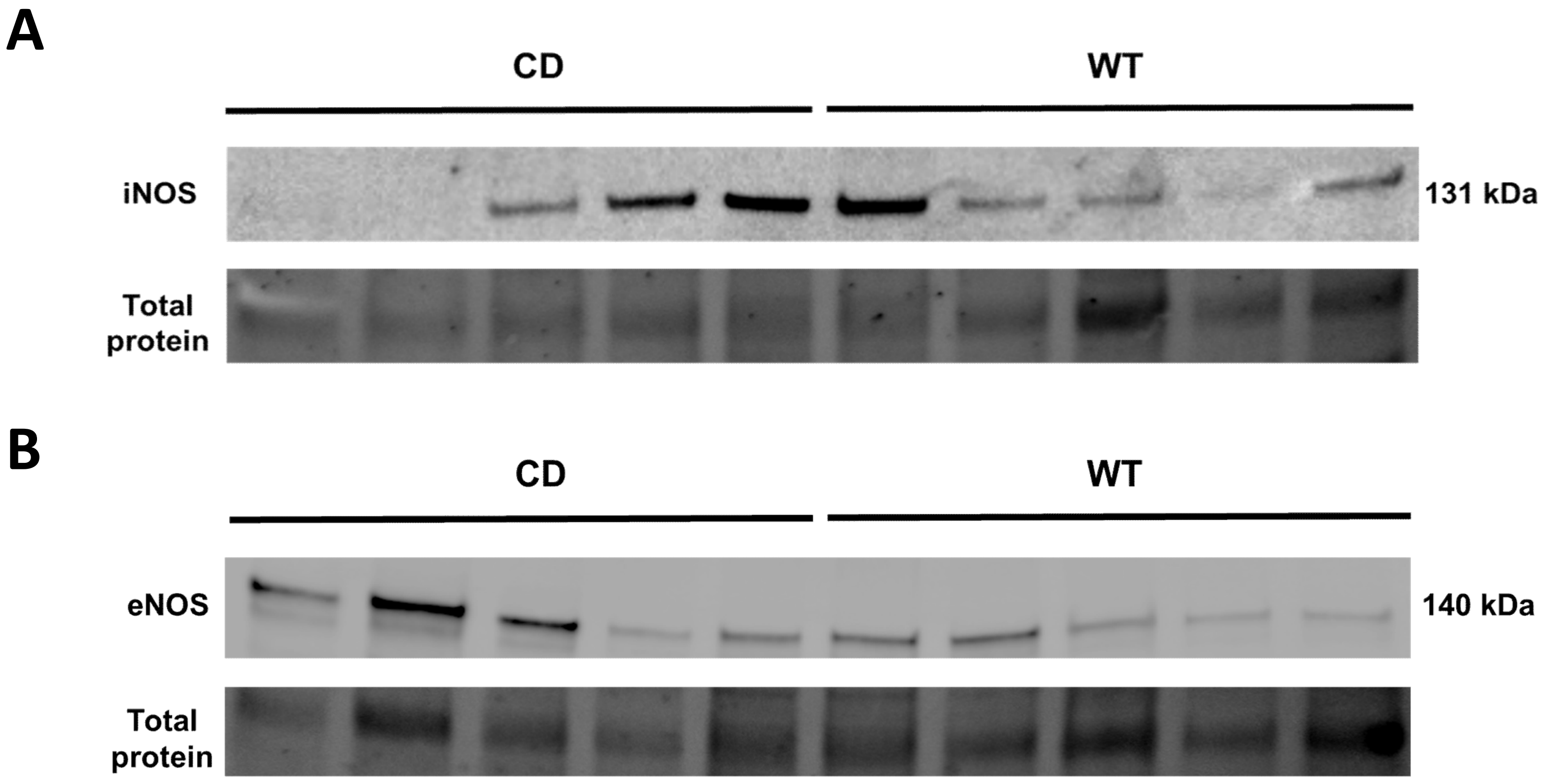

Supplementary Figure 2. Representative blots of Western blot analysis for (A) iNOS and (B) eNOS protein expression in ascending aortas from wild-type (WT) and complete deletion (CD) mice. The molecular weight (kDa) of the protein is shown on the right side of the blot. Total protein was used as a loading control.
